# Research on SPDTRS-PNN based intelligent assistant diagnosis for breast cancer

**DOI:** 10.1038/s41598-023-28316-6

**Published:** 2023-03-16

**Authors:** Xixi Kong, Mengran Zhou, Kai Bian, Wenhao Lai, Feng Hu, Rongying Dai, Jingjing Yan

**Affiliations:** School of Electrical and Information Engineering, Anhui University of Science and Technology, Huainan, 232001 China

**Keywords:** Breast cancer, Mathematics and computing, Computational science, Computer science, Statistics

## Abstract

Breast cancer is the second dangerous cancer in the world. Breast cancer data often contains more redundant information. Redundant information makes the breast cancer auxiliary diagnosis less accurate and time consuming. Dimension reduction algorithm combined with machine learning can solve these problems well. This paper proposes the single parameter decision theoretic rough set (SPDTRS) combined with the probability neural network (PNN) model for breast cancer diagnosis. We find that when the parameter value of SPDTRS is 2.5 and the SPREAD value is 0.75, the number of 30 attributes of the original breast cancer data dropped to 12, the accuracy of the SPDTRS-PNN model training set is 99.25%, the accuracy of the test set is 97.04%, and the test time is 0.093 s. The experimental results show that the SPDTRS-PNN model can improve the ac-curacy of breast cancer recognition, reduce the time required for diagnosis.

## Introduction

Breast cancer is the most common cancer diagnosed by women aged 20–60. There are more than 2 million newly diagnosed cases of breast cancer worldwide each year. Although the incidence rate of breast cancer has gradually declined in recent years to stabilize^1^, breast cancer is still one of the most common types of cancer in women, which seriously affects diseases that threaten women's life and health^2–4^. Early breast cancer is commonly identified by mammography, ultrasound, and so on. How-ever, breast cancer still nearly 30% of cases are detected in the late stage of breast cancer^5^. We can improve the success rate and reduce the mortality rate if we find breast cancer early^6,5^. Therefore, how to diagnose malignant tumors quickly and accurately is the key in the treatment of breast cancer.

The early treatment of breast cancer is needle biopsy based on tissue biopsy. In this method, a thin hollow needle into the lump to sample cells, examining the cells sampled under a microscope. But this method may lead to misdiagnosis in the process of data collection because of some uncertain factors. In addition, when the pathologist manually inspects the abnormality, their experience may affect the diagnostic results^7,8^. To solve this problem, Wolberg et al.(1994)^9^ tried to use machine learning technology to reduce the subjectivity inherent in the visual diagnosis of needle aspiration cytology. Nowadays, many algorithms in machine learning can distinguish benign and malignant breast cancer samples well and better assist in medical diagnosis^10,11^. For example, Al-Timemy et al.(2009)^12^used fine needle aspiration cytology combined with PNN to achieve rapid and accurate classification of breast tumors. Whitney et al.(2020)^13^proved the practicability of transfer learning in computer-aided diagnosis by using the breast fusion classifier based on convolutional neural network (CNN) transfer learning combined with magnetic resonance imaging (MRI). Nagpur et al.(2020)^14^ used adaptive mean, gaussian mixture model (GMM) segmentation, and probabilistic neural network (PNN) classifier to predict whether there are benign or malignant cells in a given mammogram can help patients find diseases faster and take appropriate measures. But most of these machine learning analyzed all the features contained in the breast cancer dataset. They did not consider whether the data set contains redundant information and whether the redundant information will affect the experimental results. Some dimensionality reduction algorithms and classification models were proposed to identify malignant breast tumors in Wisconsin by using Wisconsin Breast Cancer Database (WBCD)^15^. For example, Zhou et al.(2015)^16^ used principal component analysis (PCA) to preprocess the original breast cancer data and use the improved PNN model to realize the recognition of breast tumors, to achieve the auxiliary diagnosis of breast tumors. Kejriwal et al.(2018)^4^ used a univariate feature selection algorithm combined with logic and neural network algorithm to obtain a good classification effect. Bian et al.(2020)^17^ Proposed that the dimensionality reduction algorithm based on random forest (RF) and principal component analysis (PCA) combined with extreme learning machine (ELM) significantly reduced the time required for breast cancer diagnosis, showing excellent classification performance. Bashier ElKarami et al.(2022) ^18^ used the method of multi-group data integration constructed by gene similarity combination to embed gene expression, DNA methylation, and copy number alteration (CNA) into lower dimensions using UMAP to create two-dimensional RGB images. Gene expression is used as a reference for constructing GSN, and then other omics data are integrated with gene expression to better predict. Gene similarity network (GSN) Based on Unified Manifold Approximation and Projection (UMAP) and Convolution Neural Network (CNN).Li Zhou et al.(2022) ^19^ used t-distributed stochastic neighbor embedding (t-SNE) to create a gene similarity network (GSN) map for each component. Extraction of multi-group biomarkers related to the prognosis and prognosis prediction of breast cancer and establishment of prediction models for multi-category NPI of breast cancer. The model is evaluated and compared with different high-dimensional embedding techniques and neural network combinations. The accuracy of the proposed model is 98.48% better than that of other methods, and the area under the curve (AUC) is equal to 0.9999. And the findings in the literature confirmed the correlation between some extracted omics and the prognosis and survival rate of breast cancer.

The rough set theory is a novel mathematical tool for dealing with uncertain, fuzzy, and inconsistent data proposed by Professor Pawlak in 1982^20–22^. The rough set provides an effective method for multi-source heterogeneous information classification without prior knowledge^23^. The rough set theory can find the dependency between data, and reduce the number of attributes of the data set. So the rough set is widely used in dimension reduction^24^. But the classical rough set is only suitable for discrete data. So the classical rough set has been popularized from many aspects^25^. Suo M^26^ proposed a Single-parameter decision-theoretic rough set (SPDTRS), which can determine the loss function matrix by setting a single parameter and improves engineering practicability. The SPDTRS used a large number of data to prove the reliability of the model. The PNN is a feedforward neural network^27,28^, which is essentially a supervised network classifier based on the Bayesian minimum risk criterion. It has a simple structure and PNN commonly used in classification and identification^27,29^. Wang X X used PNN to intelligently classify photovoltaic array faults, achieving high classification accuracy^30^.

The main work of this paper is to use SPDTRS to reduce the dimension of breast cancer data, divide the reduced data as the input of PNN, divide the sample training set and test set. The training set is used for modeling PNN, and the test set is used to test the model. Then we can build a breast cancer classification model based on SPDTRS-PNN and optimize it. Finally, the conclusion and prospects summarize at the end of the article.

## Methods and materials

### Main contents

**Figure 1:**
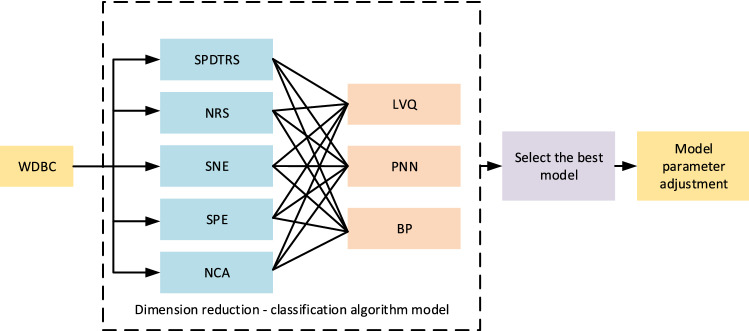
Main frame diagram.

The main framework of this paper is in Fig. 1 The main idea is to use the five-dimensional reduction algorithm and three machine learning algorithms to establish fifteen different models for distinguishing breast cancer data. We selected the better model from 15 models, the dimensionality reduction algorithm is adopted to reduce data redundancy, and the classification algorithm is adopted to classify breast cancer data. Then the selected model is optimized to achieve fast and accurate identification of breast cancer data.

### Data description

There are 32 attributes in total, including ID number and diagnostic sample label. Therefore, there are 30 attributes representing data features in each instance, including the average value, standard deviation, and maximum value of 10 quantitative features in each nucleus in the sample organization,1–10 attributes representing the average value of 10 quantitative features, and attributes 11–20 represent standard deviation of 10 quantitative features, the 21–30 attributes represent the maximum value of 10 quantitative features. Among them, the 10 quantitative features are radius, texture, perimeter, area, smoothness, compactness, concavity, concave points, symmetry, and fractal dimension. The dataset used in this paper has 569 samples in total, including 357 benign samples and 212 malignant samples.

### Selection of training set and test set

569 cases of breast cancer dataset were randomly divided into a training set and test set. 400 cases were selected as a training set, and the remaining 169 cases were taken as test sets. The computer processor used in the experiment was Intel core i3-4005U, 4 GB memory, Win7 system, and was simulated under Matlab R2014b version.

### Dimension reduction algorithm and Classification algorithm

This paper adopts the neighborhood rough set (NRS)^31^, the single-parameter decision-theoretic rough set (SPDTRS) ^26^, the stochastic neighborhood embedding (SNE)^32^, the stochastic proximity embedding (SPE)^33^and the neighbor component analysis (NCA)^34^. A suitable dimension reduction algorithm is selected as the input of the classifier.

In this paper, three classification algorithms in machine learning, the probability neural network (PNN)^28^, the learning vector quantization (LVQ)^35^, and the backpropagation (BP)^36^, are used to select the more appropriate classification algorithm.

### SPDTRS algorithm

Based on the theory of classical rough set, the probabilistic rough set proposes two threshold parameters $\alpha$ and $\beta$, in which $0 < \beta < \alpha < 1$. Decision theory rough set combines probability rough set with Bayesian minimum risk to give state set $\Omega = \left\{ {X,X^{C} } \right\}$ and action set $A = \left\{ {a_{P} ,a_{B} ,a_{N} } \right\}$, in which,$X$ represents the entity set satisfying condition $C$,$X^{C}$ represents the entity set that does not meet condition $C$, and $C$ is the conditional attribute set, $a_{P} ,a_{B} ,a_{N}$ represent three behaviors, namely, accepting events, delaying decision-making , and rejecting something, then, the loss function matrix can be described. When $X$ meets condition $C$, the losses when making $a_{P} ,a_{B} ,a_{N}$ actions are recorded as $\lambda_{PP} ,\lambda_{BP} ,\lambda_{NP}$, similarly, when $X$ does not meet condition $C$, the losses when making $a_{P} ,a_{B} ,a_{N}$ action are recorded as $\lambda_{PN} ,\lambda_{BN} ,\lambda_{NN}$, but six loss functions need to be set artificially. On this basis, SPDTRS26 sets a compensation coefficient $\xi$ to replace the six loss functions.

Given an information system $$IS = (U,A)$$, $$A$$ is the attribute set, $$A = C \cup D$$, $$D$$ is the decision attribute set, when $$C \cup D \ne \emptyset$$$$,D \ne \emptyset$$, the decision system $$DS = (U,C \cup D)$$ is defined. The inherent category represented by $$D$$ is defined as the nominal decision class, represented by $$N$$,SPDTRS gives a loss function matrix according to the property that significance represents the importance of local equivalence classes in their relevant global statistical distribution, as shown in Table 1, among them, to simplify the research, let $$\lambda_{PP} = 0$$,$$\lambda_{NN} = 0$$,$$S\left( {X|\left[ x \right]} \right)$$ represents the significance of the $$N$$-labeled sample of $$X$$ in $$\left[ x \right]$$ to $$X$$,$$S^{C} \left( {X|\left[ x \right]} \right)$$ represents the significance of the $$N$$-labeled sample of $$X^{C}$$ in $$\left[ x \right]$$ to $$X$$,and $$S\left( {X|\left[ x \right]} \right)$$ and $$S^{C} \left( {X|\left[ x \right]} \right)$$ can be obtained from the distribution information of original data, so the loss function matrix is only related to the compensation coefficient $$\xi$$.

**Table 1:** Loss function matrix.

	$X$	$X^{C}$
$a_{P}$	$\lambda_{PP} = 0$	$\lambda_{PN} = S^{C} \left( {X|\left[ x \right]} \right)$
$a_{B}$	$\lambda_{BP} = S\left( {X|\left[ x \right]} \right)\left( {P\left( {X|\left[ x \right]} \right) - \xi } \right)$	$\lambda_{BN} = S^{C} \left( {X|\left[ x \right]} \right)\left( {1 - P\left( {X|\left[ x \right]} \right) - \xi } \right)$
$a_{N}$	$\lambda_{NP} = S\left( {X|\left[ x \right]} \right)$	$\lambda_{NN} = 0$

The overall risk is defined according to Bayesian risk decision $\Re_{B}$ ^37^:

1 $$ \begin{gathered} \Re_{B} = \sum\limits_{{x \in POS^{s} }} {\left( {1 - P\left( {X|\left[ x \right]_{B}^{\delta } } \right)} \right)} \cdot \lambda_{PN} + \sum\limits_{{x \in BND^{s} }} {\left( {P\left( {X|\left[ x \right]_{B}^{\delta } } \right) \cdot \lambda_{BP} } \right.} \hfill \\ {\kern 1pt} {\kern 1pt} {\kern 1pt} {\kern 1pt} {\kern 1pt} {\kern 1pt} {\kern 1pt} {\kern 1pt} {\kern 1pt} {\kern 1pt} {\kern 1pt} {\kern 1pt} {\kern 1pt} {\kern 1pt} {\kern 1pt} {\kern 1pt} {\kern 1pt} {\kern 1pt} {\kern 1pt} {\kern 1pt} {\kern 1pt} {\kern 1pt} {\kern 1pt} + {\kern 1pt} \left. {\left( {1 - P\left( {X|\left[ x \right]_{B}^{\delta } } \right)} \right) \cdot \lambda_{BN} } \right){\kern 1pt} {\kern 1pt} {\kern 1pt} {\kern 1pt} {\kern 1pt} {\kern 1pt} + \sum\limits_{{x \in NEG^{s} }} {P\left( {X|\left[ x \right]_{B}^{\delta } } \right)} \cdot \lambda_{NP} \hfill \\ \hfill \\ \end{gathered} $$

When $B(B \subseteq C)$ satisfies the following two conditions, subset $B$ is considered to be an attribute reduction with lower risk than $C$. the conditions are as follows:

1) $$\Re_{B} < \Re_{C}$$;

2) $$\forall A \subset B,\exists \Re_{A} > \Re_{B}$$.

### PNN algorithm

A Probabilistic neural network (PNN)^38^ is a feedforward neural network extended from the nonparametric probability density estimation method based on Bayes classification rules and kernel density estimation38. PNN can use linear learning algorithms instead of nonlinear learning algorithms, and can meet the requirements of real-time processing in training.

**Figure 2:**
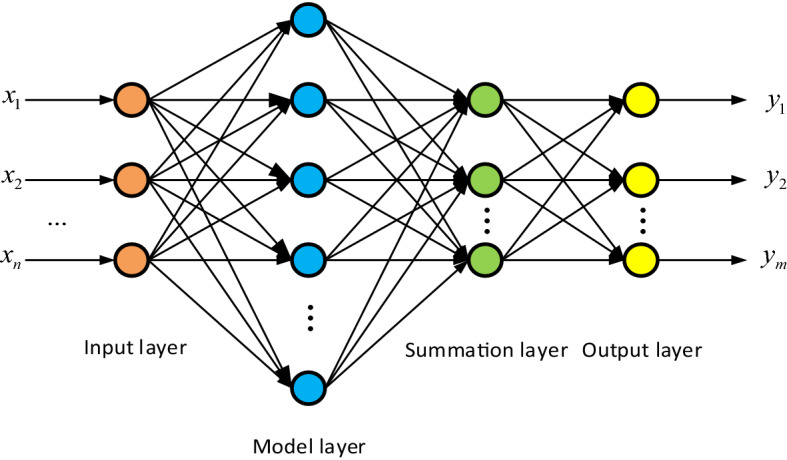
PNN structure diagram.

PNN is a four-layer feedforward neural network, as shown in Fig. 2. PNN includes the input layer, model layer, summation layer, and output layer. The input layer inputs the value of training samples and is responsible for transmitting feature vectors to the network; the model layer and input layer connection through the connection weight. Generally, the neuron activation function of the model layer is Gaussian function, which is used to calculate the similarity between the input eigenvector and each mode in the training set, and send its distance to the Gaussian function to obtain the output of the model layer; the summation layer is responsible for connecting the pattern units of each class. Each class has only one summation unit. The summation unit only adds the pattern units belonging to its class and has no connection with the pattern units of other classes; the output layer is responsible for outputting the highest score of the summation layer, and the number of output neurons is equal to the number of sample categories.

The basic structure diagram of PNN is given below:

Input layer: input the n-dimensional samples $x = [x_{1} ,x_{2} , \cdots ,x_{n} ]^{T}$ to be classified into the network;

Model layer: receive input data $$x$$, output of $$\psi_{ij} \left( x \right)$$ of $$j^{th}$$ corresponding to class $$i$$$$i^{th}$$:

2 $$ \psi_{ij} \left( x \right) = \frac{1}{{\left( {2\pi } \right)^{b/2} \delta^{b} }}\exp \left[ { - \frac{{(x - x_{ij} )^{T} (x - x_{ij} )}}{{2\delta^{2} }}} \right] $$

where $j = 1,2,3 \cdots c_{i}$, $i = 1,2,3 \cdots n$,where $c_{i}$ is the number of class $i$ training samples; $n$ is the total number of training samples; $b$ is the dimension of each sample;$x_{ij}$ is the $j$ center vector of class $i$ of the model layer; $\delta$ is the smoothing factor, which plays an important role in classification;

Summation layer: add the mode units of the same kind and calculate their average value $g_{{i,c_{i} }} \left( x \right)$:

3 $$ g_{{i,c_{i} }} \left( x \right) = \frac{1}{{c_{i} }}\sum\limits_{j = 1}^{{c_{i} }} {\psi_{ij} } \left( x \right) $$

Output layer: the $n$ outputs obtained by the summation layer are multiplied by the a priori probability $p_{i} = \frac{{c_{i} }}{n}$ of each category, and the maximum output value is the predicted test sample label value $\mu \left( x \right)$,

4 $$ \mu \left( x \right) = \arg \max \left[ {p_{i} g_{{i,c_{i} }} \left( x \right)} \right] $$

## Results

### Model selection

To obtain a better classification and recognition effect on the premise of using no more than half of the attributes ($\le$ 15), this paper tries five dimensionality reduction methods.

For SPDTRS, the compensation coefficient $\xi$ represents the tolerance of the decision-maker to uncertainty. The smaller $\xi$, the greater the amount of acceptable uncertainty. When the conservatism of the object is unknown, the value range of the compensation coefficient $\xi$ should be $(0,0.4]$. Therefore, we select the value of $\xi$ every 0.05, combined with PNN, LVQ, and BP classifiers to construct three different models of SPDTRS-PNN, SPDTRS-LVQ, and SPDTRS-BP. We compare the accuracy and test time of the training set and test set of the three models under different $\xi$ values. Taking the accuracy as the main evaluation index and combined with the test time, we select a better value of $\xi$.

The training results are in Table 2. We can see that the accuracy of the training set and test set of the three models under different $\xi$ values are more than 85.00%, which can be seen from the data in the table when the $\xi$ value of the SPDTRS-PNN model is 0.25, the accuracy of the training set is 97.00%, the accuracy of the test set is 97.04%, the test time is 0.12 s, and the training effect is good. When the $\xi$ value is 0.40, although the accuracy of the training set is 99.75%, the accuracy of the test set is only 92.90%, the accuracy is low, and the test time is 0.33 s, and the time is long, Therefore, when constructing the SPDTRS-PNN model, the value of $\xi$ is 0.25.

**Table 2:** Comparison of accuracy and time of different $\xi$ values.

SPDTRS	Dimension	PNN			LVQ			BP		
		Training set(%)	Test set(%)	Time (s)	Training set(%)	Test set(%)	Time (s)	Training set(%)	Test set(%)	Time (s)
0.05	12	97.50	94.67	0.15	90.00	88.76	0.17	98.25	95.55	0.91
0.10	12	98.50	94.08	0.14	90.00	86.98	0.12	90.15	90.07	0.86
0.15	12	97.45	93.49	0.14	90.00	88.17	0.10	95.21	95.23	0.83
0.20	12	97.25	94.67	0.13	91.00	91.02	0.10	94.71	96.62	0.85
0.25	12	**97.00**	**97.04**	**0.12**	90.00	94.08	0.10	91.27	96.98	0.94
0.30	12	98.75	94.08	0.14	87.25	86.39	0.10	**97.04**	**96.16**	**0.87**
0.35	12	99.00	92.90	0.15	87.50	86.98	0.10	91.41	90.21	0.85
0.40	14	99.75	92.90	0.33	**92.00**	**91.72**	**0.10**	95.21	96.11	0.89

The bold font in the table is the parameter value with better effect selected from each model.

When the $\xi$ value of the SPDTRS-BP model is 0.30, the accuracy of the training set is 97.04%, the accuracy of the test set is 96.16%, and the test time is 0.87 s. The training effect is good. When the value is 0.05, although the accuracy of the training set is 98.25%, the accuracy of the test set is 95.55%, and the test time is 0.91 s.

When the $\xi$ value of the SPDTRS-LVQ model is 0.40, the accuracy of the training set is 92.00%, the accuracy of the test set is 91.72%, and the test time is 0.10 s. The training effect is good. When the $\xi$ value is 0.25, although the accuracy of the test set is 94.08%, the accuracy of the training set is only 90.00%, the accuracy is low, and the test time is 0.10 s.

We can see that the SPDTRS-PNN model has a better performance by comprehensively comparing the accuracy and test time of the three models, and the number of attributes sent to the classifier after model reduction is 12 (< 15), which meets the expectation of this paper. Among them, when the value of $\xi$ is 0.25, the 12 attributes selected by SPDTRS-PNN model are {21,27,22,29,20,25,2,18,5,8,11,6}.

**Figure 3:**
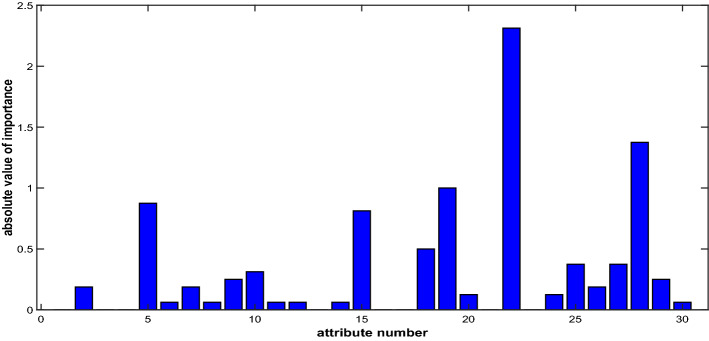
Comparison chart of absolute value of importance.

For NRS, fifteen attributes are selected according to their importance. As shown in Fig. 3, the top fifteen attributes selected according to their importance ranking are {22,28,19,5,15,18,25,27,10,9,29,2,7,26,20}.

For NRS, this paper uses the NRS algorithm to reduce breast cancer data to 1–15 dimensions and constructs three models of NRS-PNN, NRS-LVQ, and NRS-BP. We can obtain the comparison diagrams of the accuracy of the training set and the test set and test time of the three models in different dimensions.

**Figure 4:**
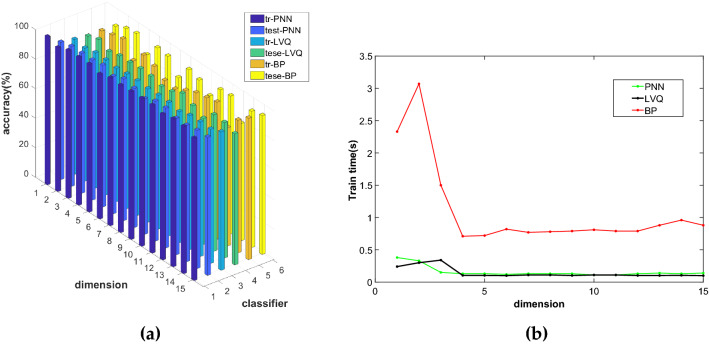
Comparison diagram of NRS-PNN, NRS-LVQ and NRS-BP models: (**a**) Accuracy comparison chart; (**b**) Comparison diagram of test time.

Figure 4a and b show the comparison of the accuracy and test time of the three models under different dimensions. It can be seen from the figure that the accuracy of the BP training set and test set is higher than LVQ and PNN, but the test time of BP is longer, and there is no difference in the test time of the other two models. Through comprehensive comparison, we can see that the NRS-BP model is better. The original data is reduced to 14 dimensions with NRS. After recognition with BP, the accuracy of the NRS-BP training set is 95.16% and the test set is 93.79%, the test time is 0.96 s.

For SNE, this paper uses the SNE algorithm to reduce breast cancer data to 1–15 dimensions and constructs three models of SNE-PNN, SNE-LVQ, and SNE-BP. We can obtain the comparison diagrams of the accuracy of the training set and the test set and test time of the three models in different dimensions.

**Figure 5:**
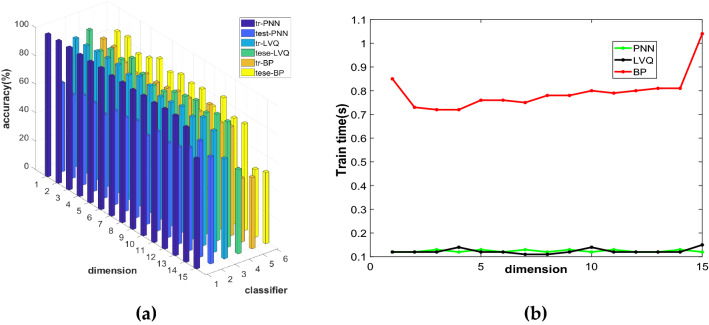
Comparison diagram of SNE-PNN, SNE-LVQ and SNE-BP models: (**a**) Accuracy comparison chart; (**b**) Comparison diagram of test time.

Figure 5a and b show the comparison of the accuracy and test time of the three models under different dimensions. It can be seen from the figure that although the accuracy of PNN's training set is higher, the accuracy of PNN's test set is lower, and there may be an overfitting phenomenon, on the whole, the training effect of LVQ is good. For PNN, the overall training effect is poor. Through comprehensive comparison, we can see that the SNE-LVQ model is better. After reducing the dimension of the original data to 15 dimensions by SNE, combined with LVQ for identification. At this time, the accuracy of the training set is 90.00%, the accuracy of the test set is 92.31%, and the test time is 0.15 s.

For SPE, this paper uses the SPE algorithm to reduce breast cancer data to 1–15 dimensions and constructs three models of SPE-PNN, SPE-LVQ, and SPE-BP. We can obtain the comparison diagrams of the accuracy of the training set and the test set and test time of the three models in different dimensions.

**Figure 6:**
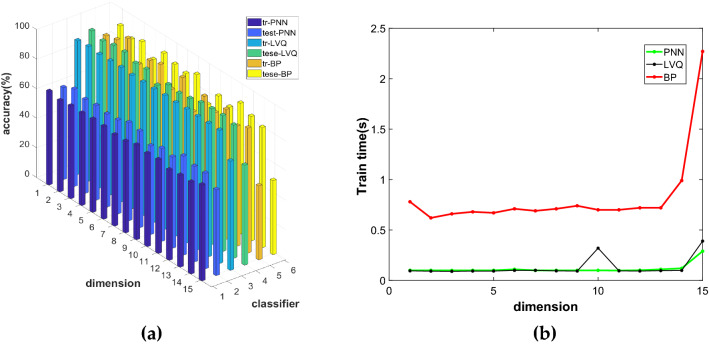
Comparison diagram of SPE-PNN, SPE-LVQ and SPE-BP models: (**a**) Accuracy comparison chart; (**b**) Comparison diagram of test time.

Figure 6a and b show the comparison of the accuracy and test time of the three models under different dimensions. It can be seen from the figure that the accuracy of the PNN training set is lower as a whole; the test time of BP is long. Through comprehensive comparison, it can be seen that the SPE-LVQ model is better. After reducing the dimension of the original data to 14 dimensions by SPE and combining LVQ for identification, the accuracy of the training set is 91.00%, the accuracy of the test set is 91.13%, and the test time is 0.099 s.

For NCA, this paper uses the NCA algorithm to reduce breast cancer data to 1–15 dimensions and constructs three models of NCA-PNN, NCA-LVQ, and NCA-BP. We can obtain the comparison diagrams of the accuracy of the training set and the test set and test time of the three models in different dimensions.

**Figure 7:**
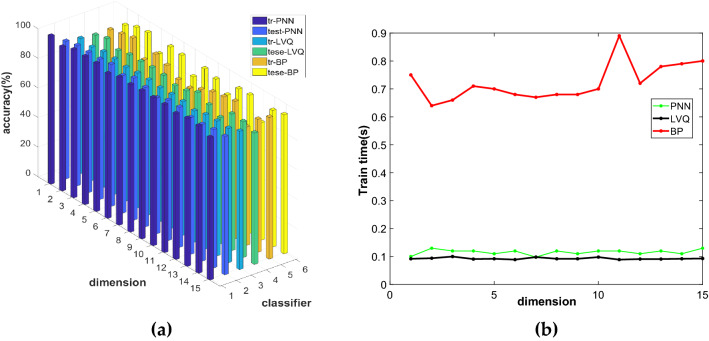
Comparison diagram of NCA -PNN, NCA -LVQ and NCA -BP models: (**a**) Accuracy comparison chart; (**b**) Comparison diagram of test time.

Figure 7a and b show the comparison of the accuracy and test time of the three models under different dimensions. It can be seen from the figure that the accuracy of PNN is higher. On the whole, the NCA-PNN model is better. After reducing the dimension of the original data to 11 dimensions by NCA, PNN is used for identification. At this time, the accuracy of the training set is 100.00%, the accuracy of the test set is 95.86%, and the test time is 0.12 s.

This paper compares the above-selected model with the accuracy and test time of putting the original data directly into the classifier, as shown in Table 3. We can see that the training effect of putting the original data into BP is the best from the table. At the same time, the accuracy of all models falls within the 95% CI for the identification and classification of any benign sample. The accuracy of the training set is 97.38% and the accuracy of the test set is 95.46%, but compared with other models, the test time is longer, the model constructed by the dimension reduction algorithm and the classification algorithm can achieve higher recognition accuracy with fewer attributes and shorten the testing time. The SPDTRS-PNN model is more accurate and the test time is 0.12 s.

**Table 3:** Comparison of different models.

Model	Dimension	Training set (%)	Training-95% CI	Test set (%)	Test-95% CI	Time (s)
PNN	30	100	[0.9804,1.0000]	72.78	[0.6623,0.8105]	0.17
LVQ	30	89.75	[0.8354,0.9154]	92.9	[0.8256,0.9440]	0.16
BP	30	97.38	[0.9578,0.9950]	95.46	[0.9094,0.9923]	1.6
SPDTRS-PNN	**12**	**97**	**[0.9235,0.9776]**	**97.04**	**[0.9041,0.9883]**	**0.12**
NRS-BP	14	95.16	[0.9453,0.9901]	93.79	[0.9041,0.9883]	0.96
SNE-LVQ	15	90	[0.8201,0.9019]	92.31	[0.8552,0.9592]	0.15
SPE-LVQ	14	91	[0.8470,0.9243]	91.13	[0.8057,0.9312]	0.099
NCA-PNN	11	100	[0.9688,1.0000]	95.86	[0.8709,0.9723]	0.12

The bold font in the table is the parameter value with better effect selected from each model.

**Figure 8:**
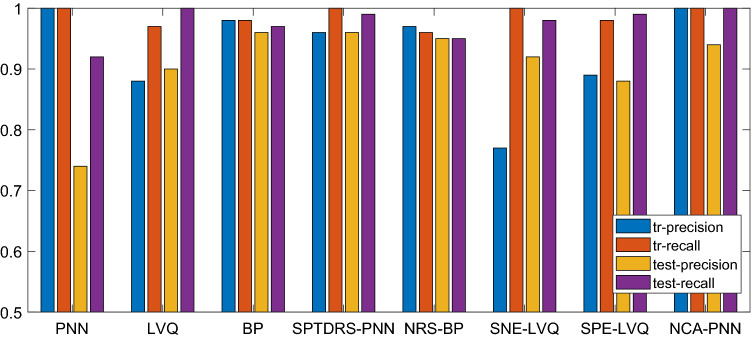
Comparison of precision rate and recall rate of each model.

The precision rate and recall rate are shown in Fig. 8. It can be seen that the precision rate and recall rate of the training set of the original data under the PNN classification are high, but the precision rate of the test set is only 74.31%. For NCA-PNN, although the precision rate and recall rate of the training set and the recall rate of the test set are high, the precision rate of the test set is low, only 93.75%; For SNE-LVQ and SPE-LVQ, the recall rate is high but the precision rate is low; For SPDTRS-PNN, the accuracy and recall of its training set and test set are more than 95%. Therefore, the SPDTRS-PNN model is selected to identify breast cancer data.

### Optimization of SPDTRS-PNN model

In this paper, we used the SPDTRS algorithm to reduced breast cancer data, and we set the SPDTRS compensation coefficient $\xi$ as 0.25. After obtaining dimension reduction, the twelve attributes are {21,27,22,29,20,25,2,18,5,8, 11,6}, and the dimensionality reduction data are taken as the input of PNN. To achieve better diagnosis and discrimination of breast cancer data, we further optimize the SPDTRS-PNN model.

For PNN, the distribution density SPREAD is the expansion coefficient of the radial basis function. Reasonably selecting the value of SPREAD is also a significant step in classification. When the value of SPREAD is close to 0, it can form the nearest neighbor classifier. When the SPREAD value is more, the output result will become smooth and can form a proximity classifier for several training samples, however, too large a SPREAD value will make numerical calculation difficult. Therefore, in this paper, the value range of SPREAD is set as $\left( {0.5,1.5} \right]$, the step size is 0.05, the accuracy is the main evaluation index, and in combination with test time to select the appropriate SPREAD parameter value.

As shown in Table 4, 569 samples were divided into 400 training samples, including 250 benign samples and 150 malignant samples; 169 test samples, including 107 benign samples and 62 malignant samples.

**Table 4:** Sample distribution table.

	Category	
	Benign	Malignant
Training set	250	150
Test set	107	62
Total	357	212

**Figure 9:**
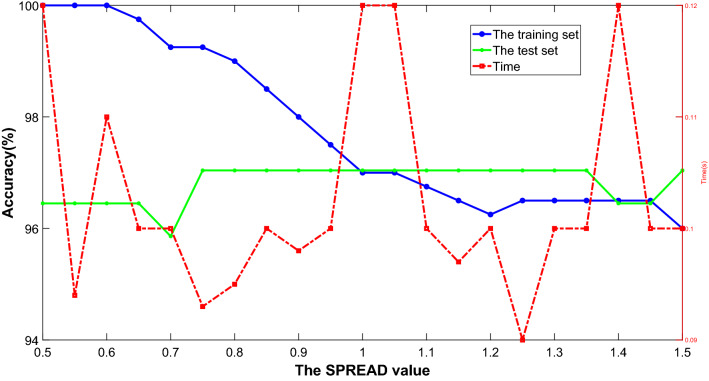
Comparison of accuracy and time of different SPREAD values.

The accuracy of the training and the test set and test time obtained under different SPREAD values are in Fig. 9. The left axis is the accuracy, the right axis is the test time, the blue solid line represents the training set accuracy, the green solid line represents the test set accuracy, and the red dotted line represents the test time. It can be seen from the figure that with the increase of SPREAD value. The accuracy of the training set of the SPDTRS-PNN model shows a downward trend as a whole. The accuracy of the test set is stable at 95.00–97.50%, and the test time fluctuates between 0.09 and 0.12 s. It can be seen from the test time curve that when the SPREAD value is 1.55, although the shortest test time is 0.09 s, the accuracy of the training set is only 96.50%, and the accuracy of the training set is low. When the SPREAD value is 0.75, the accuracy of the training set and the test set are 99.25% and 97.04%, and the test time is only 0.003 s later than the test time when the SPREAD value is 1.25. Therefore, this paper sets the SPREAD value to 0.75.

In this paper, we will use the 12 attributes of SPDTRS to reduce the dimension of breast cancer data as the input feature vector of the PNN model. The output eigenvector of PNN is the sample label, which is benign and malignant breast cancer. We set the SPREAD parameter of PNN as 0.75. A model combining SPDTRS-PNN is used to identify the breast cancer data.

**Figure 10:**
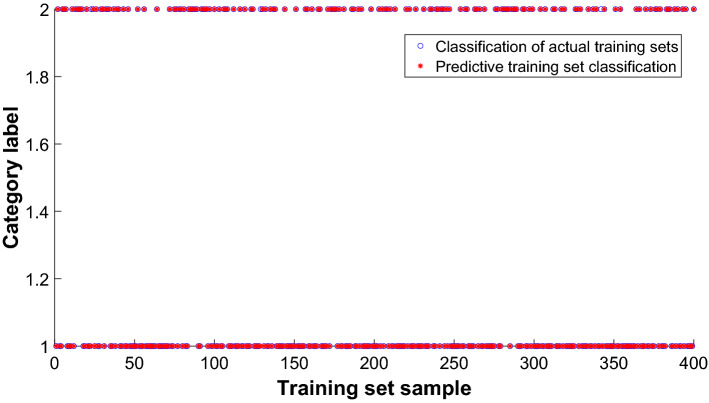
Training set classification rendering.

The 400 samples of the training set are input into PNN, and the training effect is shown in Fig. 10. The red asterisk is the output value of the network prediction, and the blue circle is the actual output value of the network. From the graph, 3 malignant tumors in the breast cancer data can be mistakenly divided into benign tumors. In general, the training set has 3 errors in the network prediction, and the accuracy rate of the training set is 99.25%, The precise is 98.81%, and the recall is 100%. For the training set, the 95% CI for any sample to judge correctly is [0.9629, 0.9969].

**Figure 11:**
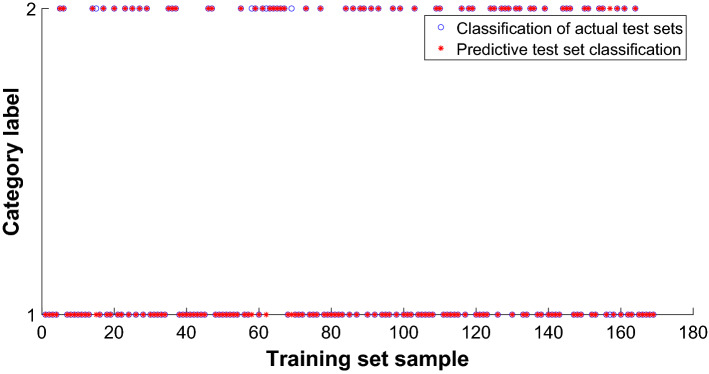
Effect drawing of test set classification.

The 169 samples of the test set were input into PNN, and the training effect is shown in Fig. 11. The red star is the output value of the network prediction, and the blue circle is the actual output value of the network. From the chart, we can see that 4 of the breast cancer data have been mistaken for benign tumors and become benign, and 1 actually benign tumor is wrongly predicted for malignant tumors. The network prediction has five errors, and the accuracy of the test set is 97.04%, The precise is 96.36%, and the recall is 99.07%. For the test set, the 95% CI for any sample to judge correctly is [0.9041,0.9883].

## Discussion

The results show that: (1) The SPDTRS can express more comprehensive information in the original breast cancer data with fewer features by selecting the appropriate value. It can reduce the complexity of the model and improves the efficiency of the model. (2) The appropriate SPREAD value was selected, then using the PNN model for classification, the test time of SPDTRS-PNN is short and fast. (3) The SPDTRS-PNN model is suitable for breast cancer diagnosis. It can objectively distinguish breast cancer from benign and malignant samples and meet the needs of a rapid and accurate breast cancer diagnosis. Although this research has made some achievements, there are still some shortcomings. To a certain extent, the SPDTRS-PNN model can reduce the redundant information of breast cancer and improve prediction accuracy. But parameters in the algorithm need artificial settings, that is, manual optimization. In future work, we need to add some automatic parameter optimization algorithms to improve the model performance and make the model performance closer to our ideal state. And when PNN is used to identify breast cancer classification, each test sample needs to be calculated with all training samples, which requires a lot of calculation. At the same time, because it needs to store all samples, the required storage space will be larger. To solve this problem, the subsequent research needs to further optimize PNN to reduce the computation and reduce the spatial complexity of the algorithm.

## Conclusions

In this paper, we combed a dimension reduction algorithm with a machine learning algorithm. Then we proposed a new auxiliary medical diagnosis method, that is, SPDTRS-PNN. The SPDTRS is used to reduce the quantitative characteristic data of breast tumor images to 12 dimensions. Then the PNN model was set up to test the predictive effect of breast cancer. We have demonstrated that the rapid and accurate diagnosis of breast cancer can be achieved by using the attributes of fewer breast cancer data.

## Data Availability

The datasets analyzed during the current study are available in the UCI repository, [http://archive.ics.uciedu/ml/datasets/Breast+Cancer+Wisconsin+%28Diagnostic%29]. And for further research in this area, we provide code on GitHub: https://github.com/kxxdget/Machine-learning-diagnosis-of-breast-cancer.

## References

[1] Marchetti P, Antonov A, Anemona L, Vangapandou C, Montanaro M, Botticelli A, Mauriello A, Gerry Melino M, Catani V (2021). New immunological potential markers for triple negative breast cancer: IL18R1, CD53, TRIM, Jaw1, LTB, PTPRCAP. Discover Oncol..

[2] Ragab DA, Attallah O, Sharkas M, Ren J, Marshall S (2021). A framework for breast cancer classification using Multi-DCNNs. Comput. Biol. Med..

[3] Basunia, M. R., Pervin, I. A., Al Mahmud, M., Saha, S. & Arifuzzaman, M. On predicting and analyzing breast cancer using data mining approach. In 2020 IEEE Region 10 Symposium (TENSYMP), 1257–1260 10.1109/TENSYMP50017.2020.9230871. (2020).

[4] Khuriwal, N. & Mishra, N. Breast cancer diagnosis using adaptive voting ensemble machine learning algorithm. In 2018 IEEMA Engineer Infinite Conference (eTechNxT), 1–5 10.1109/ETECHNXT.2018.8385355 (2018)

[5] Bhangu, K. S., Sandhu, J. K. & Sapra, L. Improving diagnostic accuracy for breast cancer using prediction-based approaches. In 2020 Sixth International Conference on Parallel, Distributed and Grid Computing (PDGC), 438–441 10.1109/PDGC50313.2020.9315815. (2020).

[6] Al-sammarraie, L. H. A. & Ibrahim, A. A. Predicting Breast Cancer in Fine Needle Aspiration Images Using Machine Learning. In 2020 4th International Symposium on Multidisciplinary Studies and Innovative Technologies (ISMSIT), 1–4 10.1109/ISMSIT50672.2020.9254891. (2020)

[7] Ahmad, F. K. & Yusoff, N. Classifying breast cancer types based on fine needle aspiration biopsy data using random forest classifier. In 2013 13th International Conference on Intellient Systems Design and Applications, 121–125 10.1109/ISDA.2013.6920720. (2013).

[8] Dennison G, Anand R, Makar SH, Pain JA (2003). A prospective study of the use of fine-needle aspiration cytology and core biopsy in the diagnosis of breast cancer. Breast J..

[9] Wolberg WH, Nick Street W, Mangasarian OL (1994). Machine learning techniques to diagnose breast cancer from image-processed nuclear features of fine needle aspirates. Cancer Lett..

[10] Thomas, T., Pradhan, N. & Dhaka, V. S. Comparative analysis to predict breast cancer using machine learning algorithms: a survey. In 2020 International Conference on Inventive Computation Technologies (ICICT), 192–196 10.1109/ICICT48043.2020.9112464 (2020).

[11] Hayashi Y (2020). Does deep learning work well for categorical datasets with mainly nominal attributes?. Electronics.

[12] Al-Timemy, A. H., Al-Naima, F. M. & Qaeeb, N. H. Probabilistic neural network for breast biopsy classification. In 2009 Second International Conference on Developments in eSystems Engineering, 101–106 10.1109/DeSE.2009.31 (2009)

[13] Whitney HM, Li H, Ji Y, Liu P, Giger ML (2020). Comparison of Breast MRI tumor classification using human-engineered radiomics, transfer learning from deep convolutional neural networks, and fusion methods. Proc. IEEE.

[14] Nagpure, R., Chandak, S. & Pathak, N. Breast cancer detection using neural network mammogram. In 2020 International Conference on Convergence to Digital World - Quo Vadis (ICCDW), 1–6 10.1109/ICCDW45521.2020.9318635 (2020)

[15] Street WN, Wolberg WH, Mangasarian OL (2012). Nuclear feature extraction for breast tumor diagnosis. Proc. SPIE.

[16] Zhou, J., Zhong, T. & He, X. Auxiliary diagnosis of breast tumor based on PNN classifier optimized by PCA and PSO Algorithm. In 2017 9th International Conference on Intelligent Human-Machine Systems and Cybernetics (IHMSC) 222–227 10.1109/IHMSC.2017.164. (2017).

[17] Bian K, Zhou M, Feng H, Lai W (2020). RF-PCA: a new solution for rapid identification of breast cancer categorical data based on attribute selection and feature extraction. Front. Genet..

[18] ElKarami B, Alkhateeb A, Qattous H, Alshomali L, Shahrrava B (2022). Multi-omics data integration model based on UMAP embedding and convolutional neural network. Cancer Inform..

[19] Zhou L, Rueda M, Alkhateeb A (2022). Classification of breast cancer Nottingham prognostic index using high-dimensional embedding and residual neural network. Cancers (Basel).

[20] Feng, Z. Q., Yun, Z. S. & Chao, B. Y. On the application of rough sets to data mining in economic practice. In 2009 International Conference on Machine Learning and Cybernetics, 272–276 10.1109/ICMLC.2009.5212452. (2009)

[21] Swiniarski RW, Skowron A (2003). Rough set methods in feature selection and recognition. Pattern Recogn. Lett..

[22] Chen Y, Chen Y (2021). Feature subset selection based on variable precision neighborhood rough sets. Int. J. Comput. Intell. Syst..

[23] Li CX, Zhen SB, Xue L, Yu HK, Qi WJ, Yan Z, Chun HQ (2020). Neighborhood rough set-based three-way clustering considering attribute correlations: an approach to classification of potential gout groups. Inform. Sci..

[24] Ping L, Heng LY (2011). Neighborhood rough set and SVM based hybrid credit scoring classifier. Expert Syst. Appl..

[25] Fan X, Zhao W, Wang C, Huang Y (2018). Attribute reduction based on max-decision neighborhood rough set model. Knowl.-Based Syst..

[26] Suo M, Tao L, Zhu B, Miao X, Zhichao Liang Y, Ding XZ, Zhang T (2020). Single-parameter decision-theoretic rough set. Inform. Sci..

[27] Naaz, S. & Parveen, S. A PNN based malign attack detection and classification model. In 2020 International Conference on Smart Electronics and Communication (ICOSEC), 933–938 10.1109/ICOSEC49089.2020.9215424 (2020)

[28] Kusy, M. Selection of pattern neurons for a probabilistic neural network by means of clustering and nearest neighbor techniques. In 2019 6th International Conference on Control, Decision and Information Technologies (CoDIT), 598–603 10.1109/CoDIT.2019.8820385. (2019).

[29] Guo J, Chen-xu G, Yang J-j, Zhang Y, Yang H (2020). Data mining and application of ship impact spectrum acceleration based on PNN neural network. Ocean Eng..

[30] Wang, X. X., Dong, L., Liu, S. Y.; Hao, Y., Wang, B. A fault classification method of photovoltaic array based on probabilistic neural network. In 2019 Chinese Control And Decision Conference (CCDC), 5260–5265 10.1109/CCDC.2019.8832338 (2019).

[31] Han, Y., Wu, X., Wu, J., Jia, R., Zhang, B. & Yao, X. A New Algorithm for Knowledge Reduction Based on Neighborhood Rough Set. In 2010 International Conference on Artificial Intelligence and Computational Intelligence, 15–18 10.1109/AICI.2010.10 (2010).

[32] Bunte K, Haase S, Biehl M, Villmann T (2012). Stochastic neighbor embedding (SNE) for dimension reduction and visualization using arbitrary divergences. Neurocomputing.

[33] Rassokhin DN, Agrafiotis DK (2004). A modified update rule for stochastic proximity embedding. J. Mol. Graph. Model..

[34] Liu C, Li X, Yang Y (2012). Text classification algorithm based on neighborhood component analysis. Comput. Eng..

[35] Sumarsono A, Supatman S (2021). Imagery identification of tomatoes which contain pesticides using learning vector quantization. J. Tek. Inform. (Jutif).

[36] Murty EM (2021). Prediksi pengadaan dan pengelolaan inventori jaringan syaraf tiruan algoritma backpropagation pada perum bulog. Komputek.

[37] Yi JX, He LW, Min TZ, Shang L (2013). Minimum cost attribute reduction in decision-theoretic rough set models. Inform. Sci..

[38] Ya, L. S., Hua, C. Z, Jing, L. & Fei, Z. Z. A medical diagnosis model based on Pnn-Cadaboost algorithm. In 2018 11th International Conference on Intelligent Computation Technology and Automation (ICICTA), 1–4 10.1109/ICICTA.2018.00008. (2018).

